# Supplementary material for: Insight into the Mechanism of Lysogeny Control of phiCDKH01 Bacteriophage Infecting Clinical Isolate of Clostridioides difficile
Source: Int J Mol Sci. 2024 May 23;25(11):5662. doi: 10.3390/ijms25115662 (PMC11172241; doi:10.3390/ijms25115662)
Supplement: Supplementary file 1 [file ijms-25-05662-s001.zip › Table S2.pdf]

Table 2 Primers used in the study

Name	5'-3' sequence
xre-RT-up	TTTTTTATGTCTGTTTTACCAAG
xre-RT-dn	CTGAGAGAAGAAAAAGGAATATC
Xre-his-up	TATAGCTAGCATGTTTAGATTAAAAGAACTG
Xre-his-dn	TATAGAATTCTAGTGATGGTGATGGTGATGTTTCTTATATTTATTCATTAAA
xre_reg_fwd	TTTGCTAGCCATATAAAATCATCCTTTATTGTAGTTTTC
xre_reg_rev	AGTGCTATCCATTTTTTCACCCTCTAACTTTTTG
GFP_fwd	CGGTACCCGGGGATCCTCTAGAGTCTTATTTGTAGAGCTCATCCATG
GFP_rev	AGGATGATTTTATATGGCTAGCAAAGGAGAAG
Crimson_fwd	AGAGGGTGAAAAAATGGATAGCACTGAGAAC
Crimson_rev	CGCCAAGCTTGCATGCCTGCAGGTCCTACTGGAACAGGTGGTG
Xre-pBAD_fwd	TTGTAAAACGACGGCCAGTGGAAGCAGGGATTCTGCAAAC
Xre-pBAD_rev	ATCCCCGGGTACCGAGCTCGTTGTAGAAACGCAAAAAGGC
S1-up	CTTCTCTCAGTTCTTTTAATC
S1-dn	GTTGCTATAATGTAAATAAAAG
S2-up	CATTATAGCAACTTTTATTTAC
S2-dn	TTATATAATAGCAACAAAAATG
S3-up	GCAACTTTTTTTGTTATTAC
S3-dn	TCACCCTCTAACTTTTTG
16S-Ec-up	CTGGACGAAGACTGACGCTC
16S-Ec-dn	GTCGACTTAACGCGTTAGCTC
Xre-bind-up	TTACAATTAAAATTGCCAAAAAAGCAACAA
Xre-bind-dn	TTGTTGCTTTTTTGGCAATTTTAATTGTAA
